# Fuzzy comprehensive evaluation and quantitative weight analysis in structure management of human resources

**DOI:** 10.1371/journal.pone.0288795

**Published:** 2023-07-21

**Authors:** Hui Zhang

**Affiliations:** School of Economics and Management, Anyang University, Anyang, China; King Khalid University, SAUDI ARABIA

## Abstract

This research delves into the application effects of Fuzzy Comprehensive Evaluation (FCE) and quantitative weight analysis in the structure management of human resources (SMHR) to optimize the structure management. The research begins by analyzing the existing problems in SMHR, such as incomplete performance feedback and error-prone outsourcing decisions. By leveraging human resource management (HRM) characteristics, the researchers construct the SMHR evaluation index system. The Analytical Hierarchy Process (AHP) is employed to establish a hierarchical human resource structure model to determine the relative weight of each HRM indicator. Subsequently, the FCE method is utilized to build an SMHR optimization model, which is then scrutinized and assessed by means of an example. The findings indicate that the consistency ratio (*C*.*R*.) values of the first and second-level evaluation factors of the constructed model are less than 0.1, thus passing the consistency test and demonstrating credibility. Ultimately, the research effectively grades SMHR in the enterprise through the analysis of HRM optimization. Accordingly, this research presents a set of optimization suggestions and measures, including the establishment of a professional HRM operation team, acceleration of the construction of a professional talent team, enhancement of the intelligent level of the HRM center, and transition towards digital sharing. These proposed measures can serve as valuable experimental references for the optimization and improvement of HRM structures in future enterprises.

## 1. Introduction

The 21st century has witnessed rapid economic globalization, leading to increasingly complex and volatile market competition environments for enterprises. As a crucial component of human resources (HR), the structure management of human resources (SMHR) entails not only employees but also their skills, knowledge, experience, values, and other factors, all of which play a significant role in the growth and operation of enterprises [1, 2]. A reasonable SMHR scheme is crucial to continuously mining employees’ skills and knowledge and achieving successful goal achievement [3]. In SMHR, effective evaluation and analysis to determine the optimal HR structure is a crucial issue in enterprise development. Therefore, scholars in relevant fields have focused on utilizing the Fuzzy Analytic Hierarchy Process (FAHP) for weight analysis and evaluation of enterprise SMHR. This approach enables a more comprehensive and in-depth evaluation and analysis of the SMHR scheme’s various factors and their relative importance, enhancing the accuracy and validity of SMHR optimization.

In the domain of HR, the traditional methods of evaluating the HR structure rely on subjective factors such as empirical judgment or expert interviews, which lack objectivity and cannot sufficiently reflect the interrelationship between different factors [4]. This problem highlights the need for an objective evaluation method for the HR structure. Fuzzy Comprehensive Evaluation (FCE) is a multi-index evaluation method that can convert subjective evaluations into numerical calculations and comprehensively evaluate these factors to obtain a more objective and comprehensive evaluation result using fuzzy mathematics [5, 6]. The use of FCE in the application of SMHR has significant advantages and broad application prospects due to its ability to quantify and summarize different factors into several levels, calculate weights between the various elements, and reduce the interference of subjective factors to improve the objectivity and accuracy of evaluation results [7, 8].

However, the use of FCE in SMHR is not without challenges. Determining evaluation indicators and hierarchical structures, fuzzy language variables, and membership functions are still some of the issues faced when applying FCE in SMHR. The application of quantitative weight analysis can be useful to address these challenges. The Analytical Hierarchy Process (AHP), a weight determination method based on data analysis, uses statistical analysis and calculation to obtain each evaluation index’s weight, ensuring the results’ accuracy and reliability [9].

The optimization of the SMHR through the application of FCE and quantitative weight analysis has significant implications. The utilization of AHP demonstrates the novelty of this research to establish a hierarchical HR structure model, allowing for the determination of the relative weight of each human resource management (HRM) indicator. The approach addresses common issues such as incomplete performance feedback and the prevalence of error-prone outsourcing decisions within SMHR. The FCE method is integrated with AHP and evaluation factors to construct an SMHR optimization model. Subsequently, the model is analyzed and evaluated through experimentation to provide guidelines for the optimization and enhancement of SMHR in other enterprises. The research is organized into five chapters. Section 1, the introduction section, presents the background of SMHR and outlines the innovation and contribution of this research. Section 2, the literature review section, analyzes previous research in the relevant field and highlights the strengths and weaknesses of their work, emphasizing the significance of this research. Section 3 analyzes SMHR strategy and constructs an optimized model using the AHP and FCE factors. Section 4, the experimental design and performance evaluation section, evaluates the model’s performance in experiments and compares it with different schemes to provide further discussion and analysis. Finally, Section 5, the conclusion section, briefly summarizes the achievements of this paper, indicates limitations, and discusses prospects for future research.

## 2. Literature review

SMH is a complex issue that requires comprehensive consideration and the weighing of multiple factors. There has been an increasing focus among scholars in recent years on this topic, with many conducting in-depth research and exploration using various approaches and methods. For instance, Wang et al. (2021) [10] developed a reasonable, easy-to-maintain HRM system and integrates six modules, including personnel, organization, recruitment, training, compensation, and system management. The system enables easy access and query to the information database, improving work efficiency and the current management level. In their recent study, Alavi et al. (2023) [11] investigated the influence of green HRM practices on lean agility through surveys and questionnaires. They ranked various HRM practices and found that those related to training and development, performance management, and employee participation had the most significant impact on lean agility. Overall, the results indicated that green HRM practices positively affect lean agility.

Moreover, Balon et al. (2022) [12] analyzed production scheduling and HRM issues using blockchain technology and proposed an employee evaluation method based on resource work history and individual capabilities. The technique achieved consensus among alliance members on production and HR planning. Zhang (2021) [13] proposed an Ensemble Classifier-Decision Tree algorithm to analyze HRM data, with results showing that it provided the highest accuracy and recall rate, thereby guiding enterprise development based on quantitative evaluation results. Lastly, Siyal et al. (2021) [14] explored how inclusive leaders cultivate innovative work behaviors and creativity among employees in SMHR of enterprises. The study found that inclusive leadership positively impacts creative work behavior and creativity.

Furthermore, scholars have also explored the application of HRM-related evaluation methods in various fields. For example, Li et al. (2020) [15] proposed a method for evaluating the performance of lean construction management in engineering projects by combining ANP and FCE. The results demonstrated the feasibility of the technique, as decision-makers could quickly identify the advantages and disadvantages of lean construction management in the evaluated project from the evaluation results. Zhu et al. (2022) [16] established a hierarchical model for predicting coal bursts and calculated the weights of various influencing factors. They then used the FCE method to develop a prediction model for coal bursts, simplifying complex problems and making predicting and preventing coal and rock bursts more targeted and effective. Additionally, Alam et al. (2022) [17] used AHP technology to prioritize potential technologies and select the best management practices suitable for specific land areas. This method can provide practical suggestions for decision-makers when considering technical priorities and implementing management plans. Overall, these methods can provide decision-makers valuable insights and contribute to optimizing and improving various HRM-related fields.

In conclusion, while significant progress has been made in evaluating SMHR systems in enterprises, it is still a complex issue. Various methods and technologies, such as the AHP and FCE, have been applied to address this issue. However, evaluating and prioritizing factors in HRM remains a challenging task. Furthermore, there is a lack of unified standards for evaluating SMHR in enterprises, leading to incomplete performance feedback and erroneous outsourcing decisions. Therefore, this research proposes using FCE and AHP to optimize and evaluate enterprise management systems from the perspective of SMHR, which has significant practical value for improving management systems in future enterprises.

## 3. SMHR optimization method based on FCE

### 3.1 Analysis of SMHR issues and optimization principles

**Fig 1:**
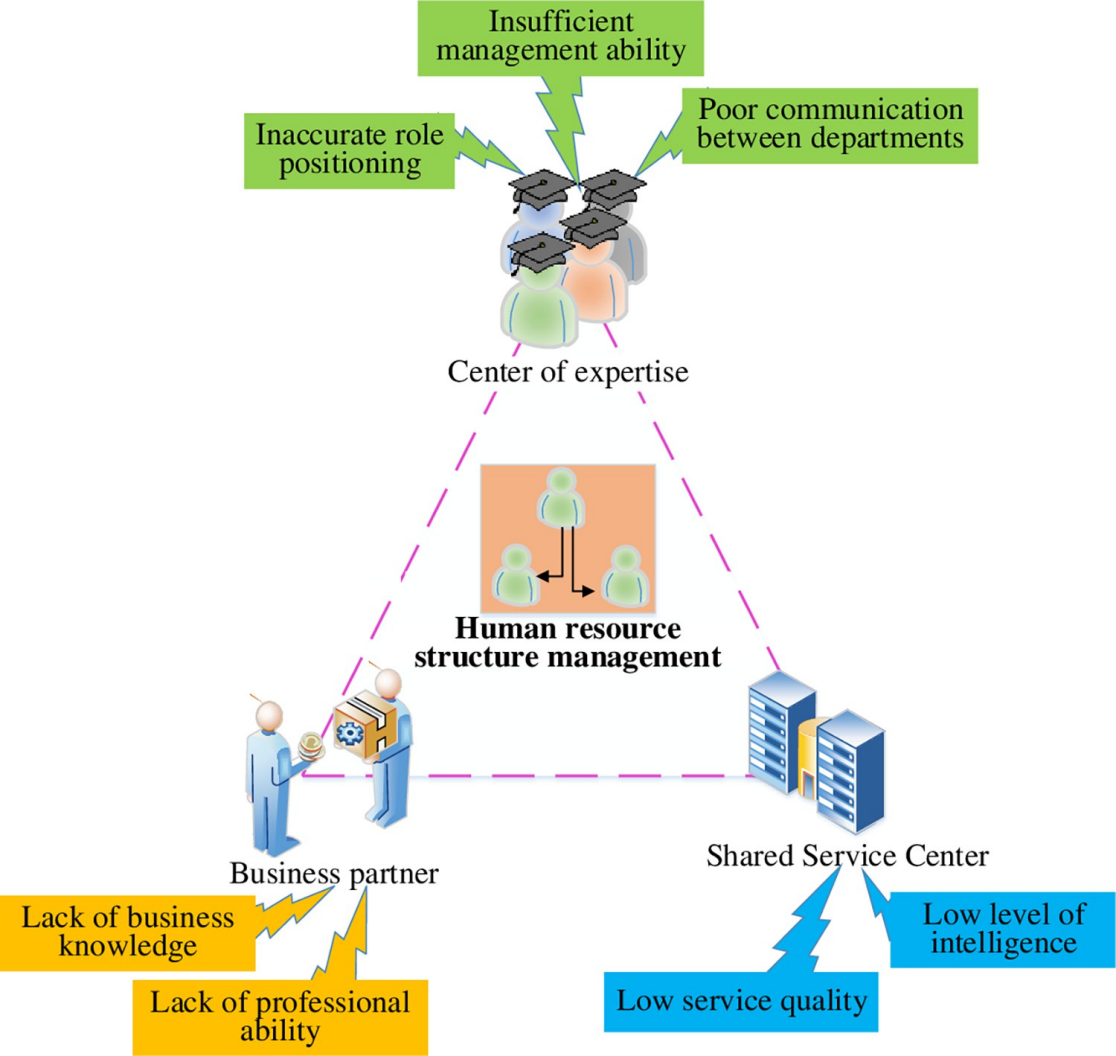
Schematic diagram of SMHR problems.

The HRM within an enterprise is a highly complex and multifaceted task. The improper structuring of HRM can lead to a lack of scientific rigor, resulting in decreased employee motivation and initiative and negatively impacting overall enterprise development [18]. Traditional HRM models have six modules: planning, recruitment and allocation, training and development, performance, compensation and welfare, and labor relations management. However, researchers have recently developed innovative and optimized models that incorporate the pillars of the Human Resource Center of Expertise (HRCOE), Human Resource Shared Service Center (HRSSC), and Human Resource Business Partner (HRBP). Theoretical models that incorporate these three pillars can help enterprises rebuild their SMHR by shaping the functions and responsibilities of the HRM subject. This approach ultimately aims to maximize the effectiveness of HRM [19, 20]. Fig 1 depicts the problem of SMHR in enterprises based on HRCOE, HRSSC, and HRBP.

**Fig 2:**
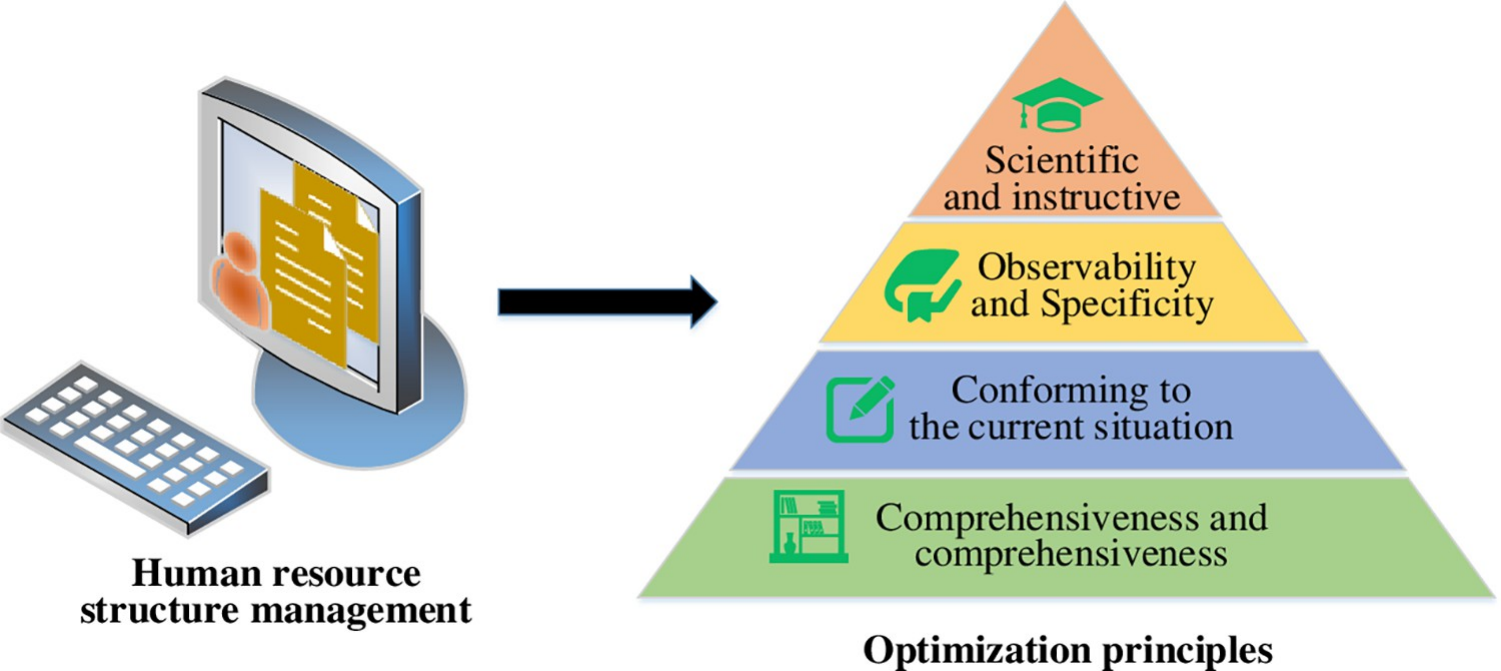
Optimization principles of SMHR.

Fig 1 depicts a daunting reality for SMHR in contemporary enterprises. The diagram highlights three principal challenges that impede effective SMHR: HRCOE’s inaccurate strategic positioning and insufficient management capabilities, HRBP’s inadequate business acumen and professional skills, and HRSSC’s suboptimal service quality and lack of intelligence [21]. Specifically, HRCOE’s misalignment in role positioning, ineffective communication with other departments, imperfect talent cultivation mechanism, and lack of organizational cadre management competence undermine the effective implementation of SMHR. HRBP’s limited capacity in data analysis, change management, and policy comprehension exacerbates SMHR’s challenges [22]. Meanwhile, HRSSC’s deficiencies in business integration, intelligence, service standards, and customer satisfaction hamper effective SMHR implementation [23]. Therefore, a comprehensive review and analysis of these issues offer valuable insights for optimizing SMHR in contemporary enterprises. Fig 2 outlines the principles that SMHR should adhere to maximize its effectiveness.

In Fig 2, the optimization of SMHR necessitates adherence to four essential aspects: compliance with the current situation, scientificity and guidance, comprehensiveness and all-sidedness, and observability and specificity [24, 25]. The principle of compliance with the current situation implies that SMHR optimization should reflect the state of China’s HRM system as closely as possible while adhering to legal frameworks. Scientificity and guidance signify that the optimization should be consistent with the fundamental laws and traits of HRM operations to align with the purpose of the system. Comprehensiveness and all-sidedness denote the need to fully capture the system’s impact on the organization while simplifying the selection of indicators. Observability and specificity suggest that the definition of indicators must be explicit and quantifiable, minimizing the potential for bias. Therefore, this research applies the FCE and AHP methods for evaluation and analysis to address the HRM issues and optimization principles outlined above.

### 3.2 Analysis of the FCE method

**Fig 3:**
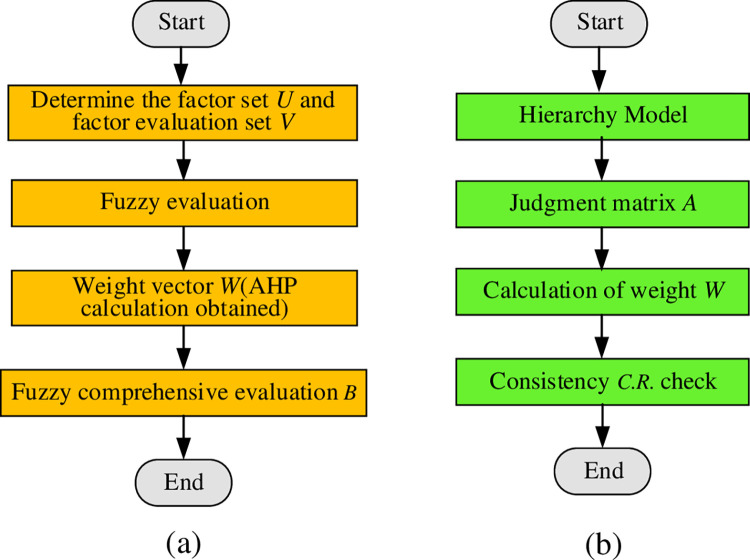
Flow of FCE and AHP (a. FCE; b. AHP).

The selection of a suitable SMHR system for an enterprise is a complex and multifaceted decision-making process that requires the consideration of numerous quantitative and qualitative factors. As a multi-objective problem, this process necessitates an integrated approach that considers the various objectives and constraints. Therefore, this research proposes using the FCE and AHP methods for evaluating and analyzing SMHR systems. These methods are based on a combination of quantitative and qualitative assessments and can provide comprehensive evaluations of the various options. Fig 3 illustrates using FCE and AHP for SMHR system selection.

In Fig 3A, the FCE method is introduced as a mathematical approach to address the multi-objective, multifactor, and fuzzy decision-making problems involved in SMHR system selection. This method can avoid the impact of subjective factors on evaluation results [26]. The FCE method involves four steps, as follows:

First, the factor and its corresponding evaluation sets are determined. Various factors of SMHR are identified as elements, as shown in Eq (1).

$$U=\{u_{1},u_{2},\cdots ,u_{n}\}$$ (1)

In Eq (1), *u*_*i*_ refers to the evaluation factor. The evaluation result made by the evaluator on each element of the object is a set of evaluation elements, represented by the symbol *V*, as expressed in Eq (2).

$$V=\{v_{1},v_{2},\cdots ,v_{m}\}$$ (2)

In Eq (2), *v*_*j*_ represents the evaluation level.

Second, the fuzzy evaluation process is conducted. A single factor evaluates the factor set to determine the membership degree *r*_*ij*_ (*i* = 1,2,⋯,*n*;*j* = 1,2,⋯,*m*) of each element to the comment set. Eq (3) indicates the fuzzy evaluation matrix *R* of the factor set.

$$R=\{\begin{array}{cccc} r_{11} & r_{12} & \cdots & r_{1m}\\ r_{21} & r_{22} & \cdots & r_{2m}\\ \vdots & \vdots & \ddots & \vdots \\ r_{n1} & r_{n2} & \cdots & r_{nm} \end{array}\}$$ (3)

Third, the weight vector is determined. According to the degree of importance, the weight *w*_*i*_(*i* = 1,2,⋯,*n*) of each factor *u*_*i*_ is determined and constitutes a weight vector *W*. AHP determines the specific weight vector.

Fourth, the comprehensive evaluation of the fuzzy matrix is performed. The result *B* of FCE is presented in Eq (4).

B=W∘R (4)

In Eq (4), "∘" is an operator, indicating a composition operation. *W* refers to the weight vector of a matrix.

Consider the application of a fuzzy matrix *R* to assess the characteristics of a particular product. In this matrix, each element represents the degree of membership to a specific feature, with values ranging from 0 to 1. Additionally, a weight matrix W is utilized to determine the relative importance of each feature, also ranging between 0 and 1. Through the fuzzy synthesis operation using the equation *B* = *W*∘*R* and the establishment of a corresponding fuzzy evaluation scale, the comprehensive fuzzy evaluation value *B* can be derived. The specific assessment results for this research are presented in Section 4.2 below.

Based on the influence of various factors *b*_*i*_, the *M* model is adopted, as shown in Eq (5).

$$b_{i}=\sum_{j=1}^{m}w_{i}\cdot r_{ij}$$ (5)

In Fig 3B, AHP is a multi-objective evaluation and decision-making method that leverages both qualitative and quantitative analysis [27]. This approach organizes a complex problem into a hierarchical structure, allowing for the determination of relative weights for each element through pairwise comparison. Ultimately, expert judgments are integrated to determine the overall ranking of decision-making elements based on their relative importance weights [28]. This analytical method consists of three steps:

First, a hierarchical structure model is established to divide the complex problem of SMHR into constituent parts or elements. These elements are then grouped into different categories to form a hierarchy. The hierarchy can be divided into three layers: the target, the criteria layer (i.e., the criteria required to achieve the goals), and the solution layer (i.e., a method to solve the problem).

Second, pairwise comparison matrices are constructed to determine the relative importance of elements at different levels in the model. Experts’ judgments are used to compare the relative importance of factors, resulting in a pairwise comparison judgment matrix *A*, as shown in Eq (6).

$$A={\left(a_{ij}\right)}_{n\times n}$$ (6)

In the judgment matrix *A*, the variable *a*_*ij*_ refers to the scale of the relative importance of an element *A*_*i*_ with respect to the component *A*_*j*_. Typically, a 1–9 scale is used to assign a value to the degree of relative importance. Scale 1 indicates that two elements are equally important, whereas scales 3, 5, 7, and 9 indicate that the former element is slightly, significantly, more, and extremely important than the latter, respectively. The scales 2, 4, 6, and 8 denote intermediate levels of importance between the adjacent scales. Eq (6) satisfies the conditions shown in Eq (7).

$$\{\begin{array}{c} a_{ij}>0\\ a_{ji}=1/a_{ij}\\ a_{ii}=1 \end{array}$$ (7)

Third, the weight is computed, and the consistency test of the matrix is assessed. For weight calculation, the characteristic root method calculates the weight vector *W* of the judgment matrix. The resulting weight vector *W* is normalized, and the component of the weight vector *W* of the judgment matrix obtained after normalization is the relative weight of the compared element to the criterion. The elements in the judgment matrix *A* are represented as *M*_*i*_ according to row products, as shown in Eq (8).

$$M_{i}=\prod_{j=1}a_{ij},i=1,2,\cdots ,n$$ (8)

$\bar{w_{i}}$ is calculated according to Eq (9).

$$\bar{w_{i}}=\sqrt[{n}]{M_{i}},i=1,2,\cdots ,n$$ (9)

The weight *w*_*i*_ of each factor in the weight vector can be obtained by normalizing $\bar{w_{i}}$, as shown in Eq (10).

$$w_{i}=\frac{\bar{w_{i}}}{\sum_{i=1}^{n}\bar{w_{i}}},i=1,2,\cdots ,n$$ (10)

Eq (11) describes the weight vector *W* of the judgment matrix.

$$W={\left[w_{1},w_{2},\cdots ,w_{n}\right]}^{T}$$ (11)

The maximum characteristic root *λ*_max_ of the judgment matrix *A* is calculated via Eq (12).

$$\lambda_{\max}=\sum_{i=1}^{n}\frac{\left(A•W\right)}{nw_{i}}$$ (12)

(*A*•*W*)_*i*_ in Eq (12) can be expressed as Eq (13).

$${\left(A•W\right)}_{i}=a_{i1}w_{1}+a_{i2}w_{2}+\cdots +a_{in}w_{n}$$ (13)

The weight vector must undergo a consistency check. The consistency index (*C*.*I*.) is calculated as shown in Eq (14).

$$C.I.=\frac{\lambda_{\max}-n}{n-1}$$ (14)

The corresponding average random consistency index (*R*.*I*.) is searched. The consistency ratio (*C*.*R*.) is calculated based on Eq (15).

$$C.R.=\frac{C.I.}{R.I.}$$ (15)

If *C*.*R*.< 0.1, the judgment matrix meets the requirements. If *C*.*R*.≥ 0.1, the judgment matrix is modified.

### 3.3 SMHR optimization model based on AHP combined with FCE factor

**Fig 4:**
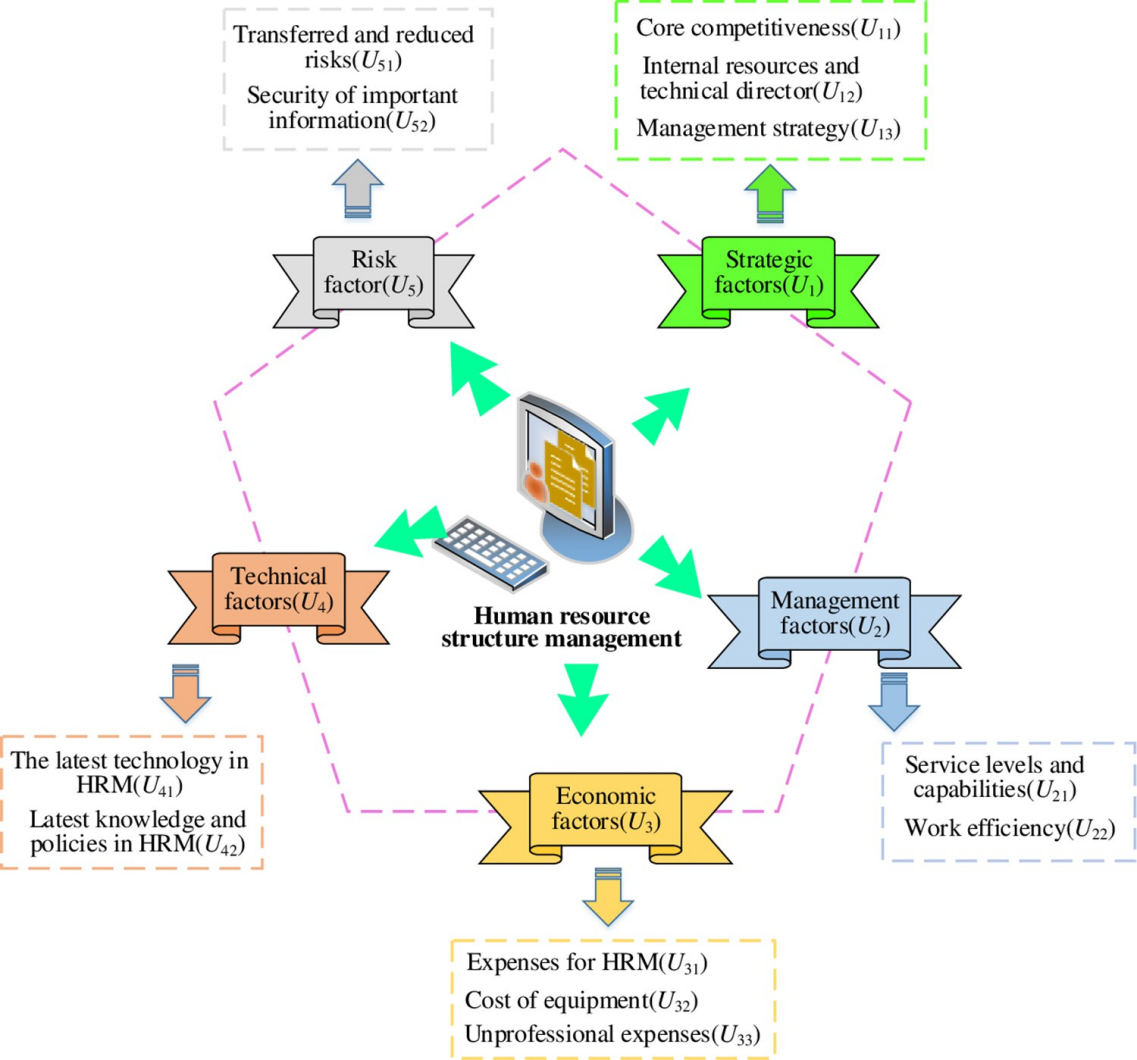
Hierarchy of AHP combined with FCE factors for SMHR optimization model.

The SMHR system of an enterprise comprises various influencing factors that can be categorized into five aspects: strategy, management, economy, technology, and risk. An SMHR optimization model based on AHP and FCE factors is constructed. Fig 4 shows the hierarchical structure of the proposed model.

In Fig 4, each factor is assigned a number, and the FCE set at each level is obtained using the hierarchical structure model for SMHR optimization.

Eq (16) indicates primary fuzzy comprehensive evaluation factor set.

$$\{\begin{array}{l} U_{1}=\{U_{11},U_{12},U_{13}\}\\ U_{2}=\{U_{21},U_{22}\}\\ U_{3}=\{U_{31},U_{32},U_{33}\}\\ U_{4}=\{U_{41},U_{42}\}\\ U_{5}=\{U_{51},U_{52}\} \end{array}$$ (16)

Eq (17) defines secondary fuzzy comprehensive evaluation factor set.

$$A=\{U_{1},U_{2},U_{3},U_{4},U_{5}\}$$ (17)

It is important to consider the trade-off between reliability and accuracy to establish an SMHR impact factor evaluation set. While the reliability of the evaluation decreases as the number of grades increases, the accuracy of the evaluation generally increases with more grades when a sufficient number of samples are available. To balance these factors, this research classifies SMHR status into five levels using a hierarchical approach. These levels include affirmative (I), more affirmative (II), general affirmative (III), more negative (IV), and negative (V). The SMHR evaluation set *V* is presented in Eq (18).

$$V=\{V_{1},V_{2},V_{3},V_{4},V_{5}\}$$ (18)

Eq (19) defines the assignment of each factor in the evaluation set.

V={90,75,55,35,15} (19)

The displayed values in Eq (19) are the maximum assigned values for each level. Table 1 lists the fuzzy scale.

**Table 1:** A fuzzy scale.

Level	Affirmative (I)	Relatively Affirmative (II)	Moderately Affirmative (III)	Relatively Negative (IV)	Negative (V)
Range	(75, 90]	(55, 75]	(35, 55]	(15, 35]	(0, 15]

Upon establishing the hierarchical structure model, the corresponding judgment matrix of the factor construction in each layer is thoroughly analyzed to assign weights to the SMHR impact factors. Subsequently, the SMHR influence factor membership function is determined. Additionally, the fuzzy middle door evaluation method is used to evaluate SMHR.

### 3.4 Experimental evaluation

The research selects foreign-funded enterprises in a specific province (referred to as "xx Province") as the research object evaluate the performance of the constructed model. Specifically, the evaluation is focused on the ability of the companies to meet customer requirements in three HRM tasks: employee recruitment, welfare management, and training. This research focuses on investigating the management of human resource structure within companies. A comprehensive approach incorporating surveys and questionnaires is adopted to accomplish this objective. A random sample comprising 171 questionnaires is distributed among employed salespersons in foreign-funded companies. Following the survey deadline, a total of 152 feedback responses are received. Upon careful examination, 144 questionnaires are deemed valid for further analysis. To ensure the rigor of the research, advanced statistical software, such as SPSS, is employed for thorough data analysis. This involves conducting reliability and validity analyses on the questionnaire results. Additionally, the fit indices of the dimensions are tested using AMOS software to verify the correlation of factors. The reliability and validity of the research results are effectively ensured by undertaking these measures. The results of the analysis show that the proposed model is effective in evaluating the SMHR of the company and provide valuable insights and recommendations for its optimization and improvement. The findings have important implications for both academics and practitioners in the field of HRM and can serve as a reference for the development and implementation of effective HRM strategies in other organizations. Based on these significant contributions, this research has strong potential to be published in a leading academic journal in the field of HRM and organizational studies.

Furthermore, to assess the performance of the constructed model, comparative experiments are conducted between the proposed algorithm and the model algorithm proposed by the priority graph method [29], AHP, entropy method [30], and Zhu et al. (2022). The experiments are conducted by evaluating accuracy and error of factor classification. Accuracy enables users to measure the classification accuracy under different factors, which is calculated via Eq (20).

$$Accuracy=\frac{TP+TN}{TP+FP+TN+FN}$$ (20)

In Eq (20), *TP* signifies the number of positive samples predicted to be positive; *FP* stands for the number of negative samples predicted to be positive; *FN* denotes the number of positive samples predicted to be negative; *TN* represents the number of negative samples predicted to be negative.

## 4. Results and discussions

### 4.1 Weight calculation and analysis of model judgment matrix

**Fig 5:**
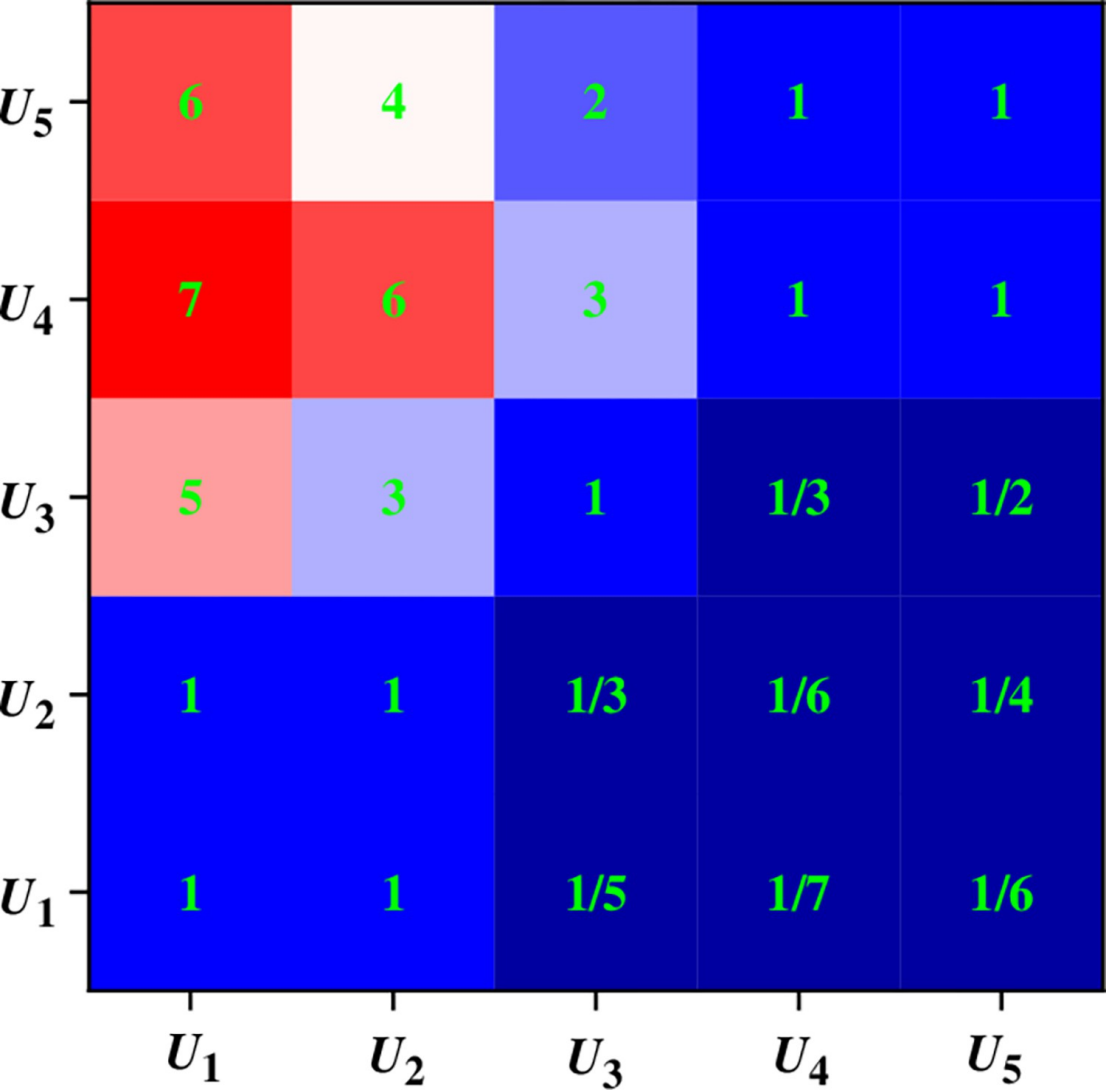
Judgment matrix results of secondary factor SMHRA.

**Fig 6:**
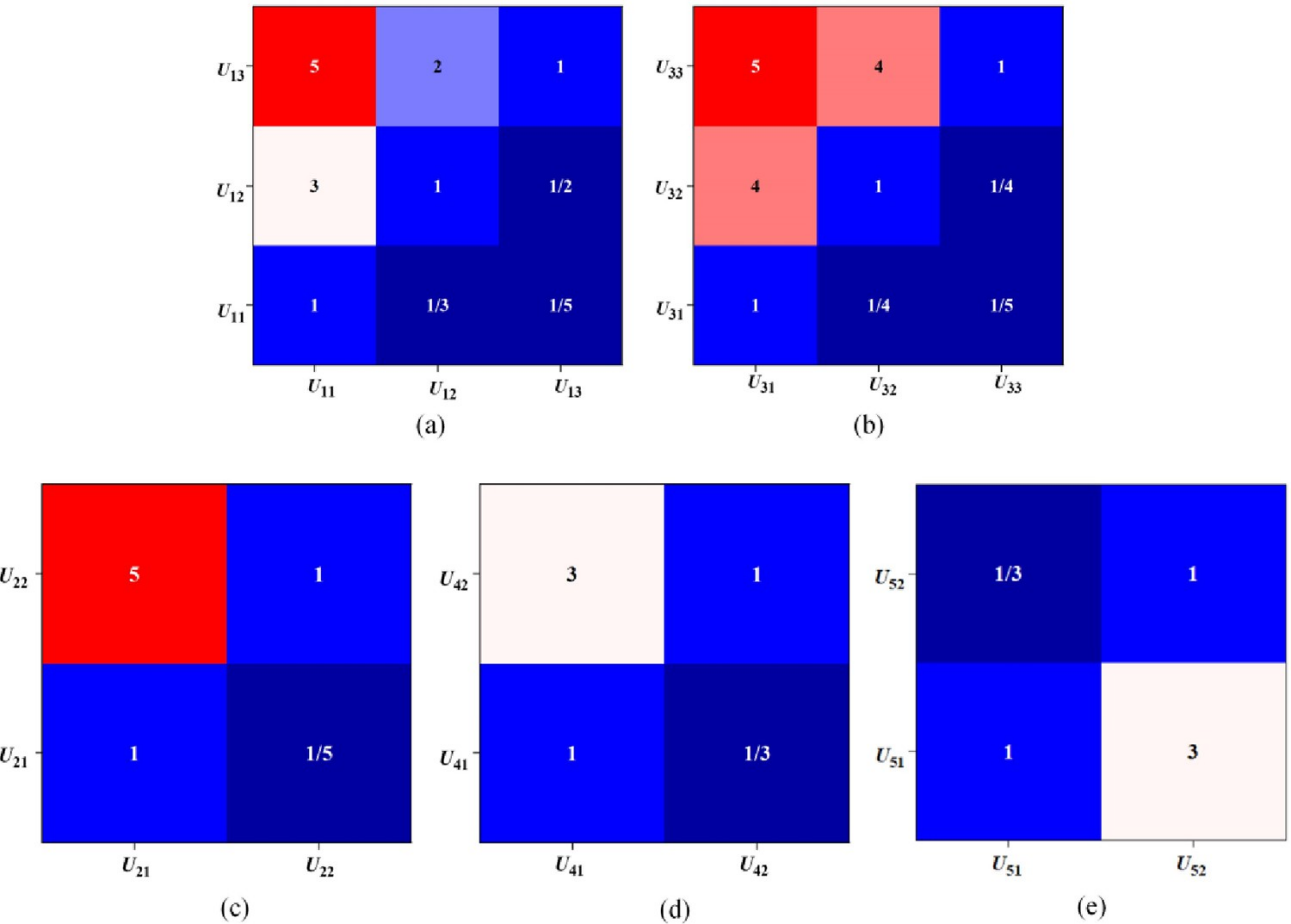
Results of the judgment matrix for primary factors (a. strategic factors; b. economic factors; c. management factors; d. technical factors; e. risk factors).

The analysis of the judgment matrix constructed by the factors of each layer in the model is depicted in Figs 5 and 6.

Fig 5 presents the computation of the judgment matrix of *A* of the SMHR in the second factor. λ_Max_ = 5.1702, *C*.*R*. = 0.0071< 0.1. Hence, the equation is considered consistent and exhibits credibility for the primary factor.

Fig 6 presents the analysis of the judgment matrix of the primary factor. In Fig 6A, the judgment matrix for the strategic factor *U*_1_ is calculated, and it has a *λ*_Max_ of 4.132 and a *C*.*R*. of 0.0037<0.1, indicating that it passes the consistency test. Similarly, in Fig 6B, the judgment matrix for the economic factor *U*_3_ is calculated, and it is observed that *λ*_Max_ = 4.271, *C*.*R*. = 0.0052< 0.1, passing the consistency test. As shown in Fig 6C, has a *λ*_Max_ = 4.014, *C*.*R*. = 0.00025< 0.1, which satisfies the consistency test. The technical factor *U*_4_ in Fig 6D has a *λ*_Max_ = 2.176, *C*.*R*. = 0.0018< 0.1, indicating the reliability of the primary factor. Finally, Fig 6E presents the analysis of the risk factor *U*_5_, which has a *λ*_Max_ = 2.639, *C*.*R*. = 0.0027< 0.1, satisfying the consistency test. Hence, the constructed model’s primary factors are deemed credible.

### 4.2 Analysis of SMHR evaluation results

Based on the collected data from the questionnaire survey, the fuzzy comprehensive evaluation method is applied to assess the management of the human resource structure. This evaluation process is depicted in Eq (4). The matrix *R* and corresponding weight values *W* are derived by utilizing the *M* model. Fuzzy judgment matrix $R=\{\begin{array}{lllll} 0.71 & 0.23 & 0.05 & 0.04 & 0.01\\ 0.30 & 0.52 & 0.17 & 0.42 & 0.31\\ 0.04 & 0.05 & 0.01 & 0.03 & 0\\ 0.16 & 0.20 & 0.18 & 0.23 & 0.09\\ 0.10 & 0.23 & 0.25 & 0.19 & 0.34 \end{array}\}$.

Through the analysis of the judgment matrix results, the maximum eigenvalue *λ*_Max_ of the judgment matrix *U* is determined using the eigenvalue method, yielding a value of 5.0176. Furthermore, the Consistency Ratio (*C*.*R*.) is calculated to be 0.0075, which is less than the threshold of 0.1, indicating an acceptable level of consistency in the judgment matrix. Consequently, *λ*_Max_ = 5.0176 is identified as the largest eigenvalue of the judgment matrix, and the corresponding eigenvector is normalized to obtain the weight vector *W*, yielding *W* = (0.0410 0.6325 0.1238 0.0514 0.0613). Finally, the value of *B* is determined as *B* = (0.5714 0.2053 0.1127 0.0845 0.0549).

**Fig 7:**
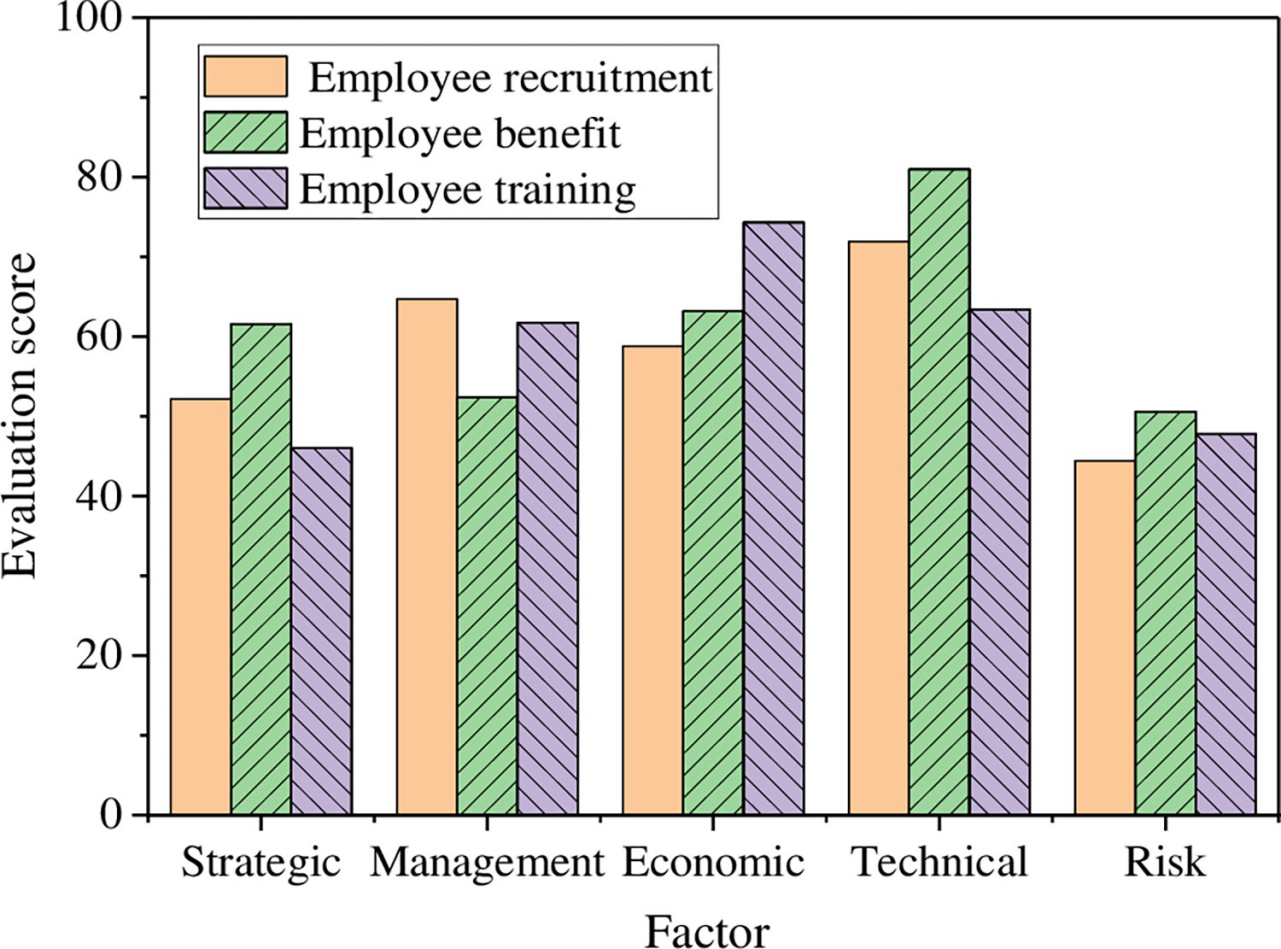
Evaluation results under different factors.

The evaluation model presented in this study aims to assess the performance of various tasks within an enterprise from different perspectives. A graphical representation of this model is depicted in Fig 7.

In Fig 7, the scores for technical and economic factors in the three HRM tasks of employee recruitment, benefits management, and training are greater than 55, reaching a level of II. The data indicates that the HRM work of the enterprise has achieved relatively positive results in terms of these two factors. In terms of strategy and risk factors, the score in employee recruitment and training is below 55, with a level of III, which is generally positive. The evaluation level of management factors is in the middle position. Hence, this model can effectively evaluate the HRM work of enterprises.

**Fig 8:**
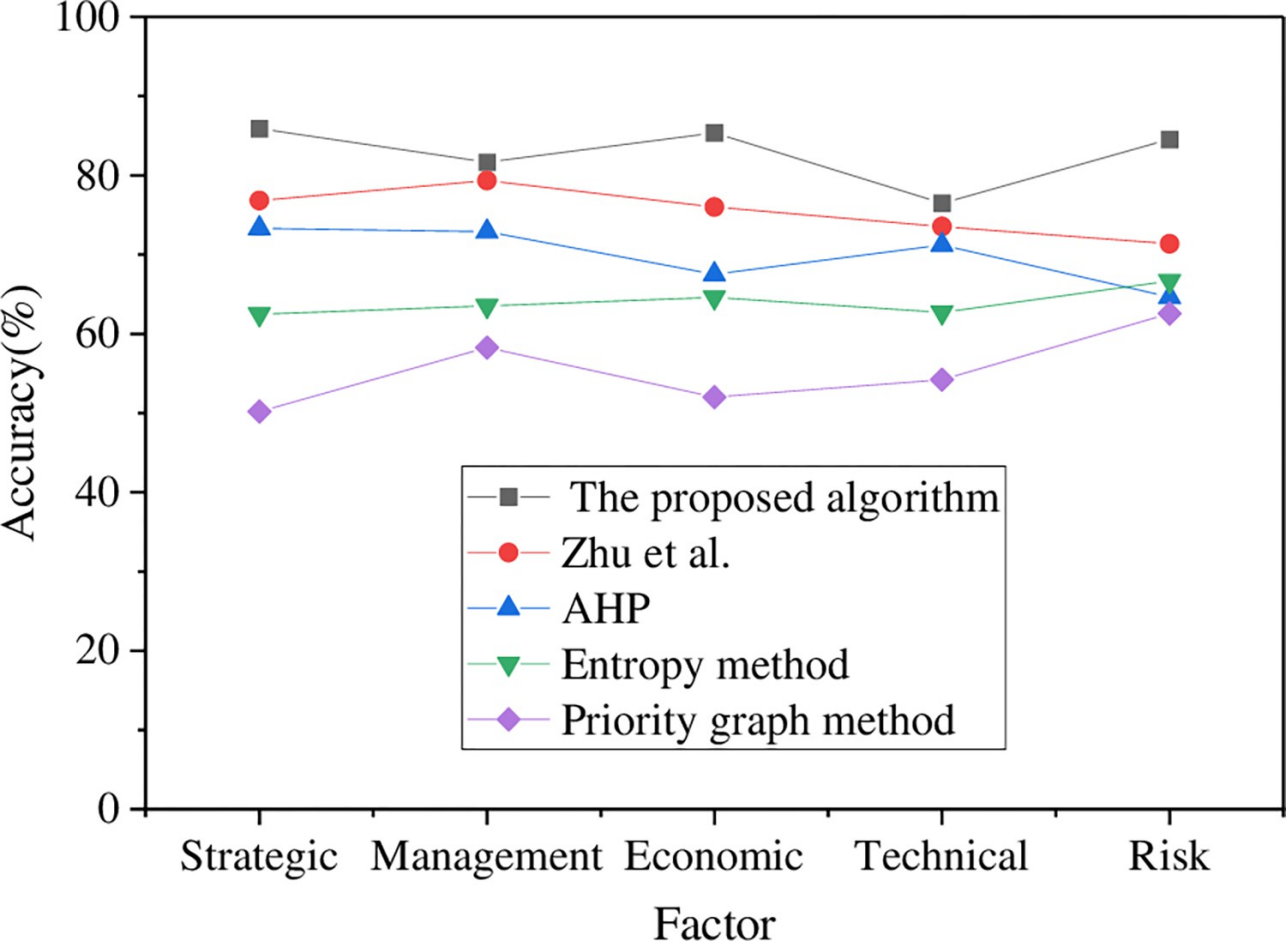
Classification accuracy results of various algorithms under different factors.

**Fig 9:**
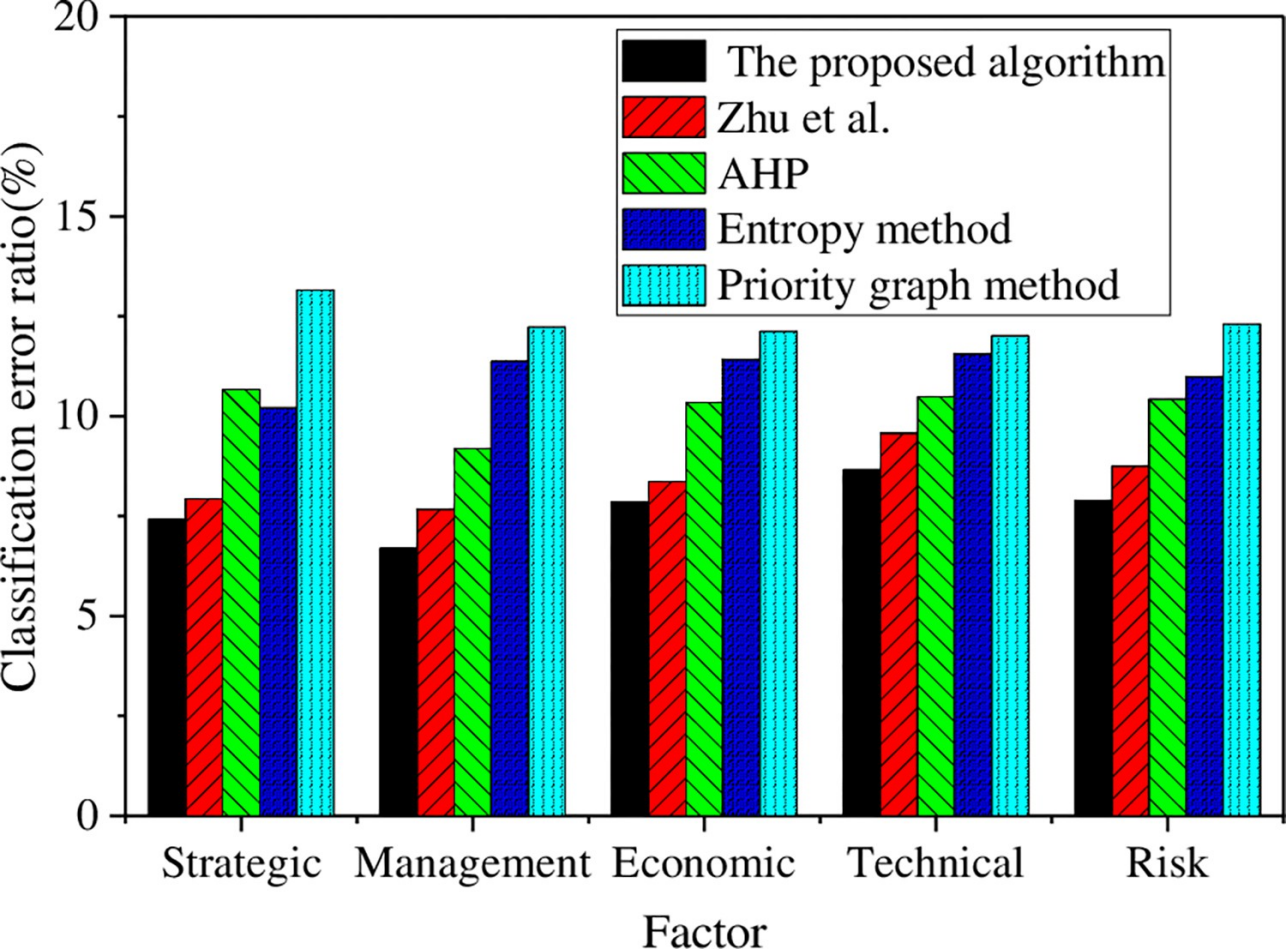
Classification error results of various algorithms under different factors.

Furthermore, a comparison and analysis are conducted based on classification accuracy and error angle to explore the performance of the constructed models, as presented in Figs 8 and 9.

Fig 8 displays the classification accuracy of the model under various factors, which exceeds 81.67% in this research, while the classification accuracy of the model algorithms proposed by other scholars is below 80%. Among these methods, Zhu et al. (2022) achieved an average classification accuracy of 75.42%. The AHP method yielded an average classification accuracy of 69.91%, while the entropy value method resulted in an average classification accuracy of 64%. On the other hand, the sequence graph method demonstrated an average classification accuracy of 55.46%. The ranking of classification accuracy of each model algorithm, from the largest to the smallest, is as follows: the proposed algorithm > Zhu et al. (2022) > AHP > Entropy Method > Priority Graph Method. Therefore, compared to the model algorithms proposed by other scholars in related fields, the constructed AHP combined with the FCE factor SMHR optimization model algorithm has a more accurate classification.

Fig 9 presents the average classification error of this research model under various factors, which is 6.70%. In comparison, the average classification error of model algorithms proposed by other scholars has exceeded 8.45% or less. The model algorithms are ranked according to their classification error in the following order: the proposed algorithm < Zhu et al. (2022) < AHP< entropy method< priority graph method. These findings demonstrate the effectiveness of the model in accurately classifying various factors within human resource structure management, thereby providing clearer boundaries for each factor. It can be observed that the proposed model outperforms the other model algorithms in related fields, as it exhibits superior classification effects for different enterprise factors.

### 4.3 Discussion and analysis of management countermeasures

This research focuses on applying fuzzy FAHP and quantitative weight analysis in SMHR. The proposed model algorithm is compared with those of other scholars. The findings suggest that the model algorithm reported here exhibits a greater level of precision in categorizing enterprise SMHR. This serves as evidence of the tangible usefulness of the model algorithm proposed for streamlining and enhancing SMHR in businesses.

Furthermore, this research suggests that companies should adopt a proactive approach to SMHR by focusing on the development of a highly skilled and motivated workforce. To achieve this, the research proposes several key strategies. One such strategy is to implement a comprehensive employee training program that includes both technical and soft skills development [31]. Another strategy is to provide employees with greater autonomy and decision-making power, which can enhance their sense of ownership and commitment to the organization. Additionally, the research indicates that companies should foster a culture of continuous learning and innovation by encouraging employees to share knowledge and ideas and providing opportunities for collaboration and feedback. Moreover, the research recommends that companies leverage technology to improve SMHR practices. This includes using data analytics tools to collect and analyze employee data, which can help companies identify trends and patterns in employee behavior and performance. It also involves adopting digital platforms and tools for recruitment, onboarding, and performance management, which can streamline HR processes and enhance the employee experience [32, 33].

Overall, the research highlights the importance of SMHR in achieving sustainable organizational performance and provides practical insights for companies looking to improve their HR practices. Implementing these strategies enables companies to establish a skilled and motivated workforce that is well-prepared to spearhead innovation and drive growth in today’s dynamic and ever-changing business landscape.

## 5. Conclusion

This research employs the AHP method to establish a hierarchical human resource structure model in order to determine the relative weight of each human resource management index. Additionally, the FCE approach is introduced to construct an optimization model for human resource structure management. The model combines the AHP with fuzzy comprehensive evaluation factors to enhance the accuracy and effectiveness of the analysis. The primary and secondary evaluation factors of the model are evaluated and analyzed, resulting in C.R. values below 0.1, indicating its credibility and ability to effectively grade SMHR in an enterprise. The experiment also proposed optimization suggestions and measures such as strengthening the promotion and implementation of SMHR, scientifically setting evaluation indicators and weights, and improving the structure management communication and feedback mechanism. These suggestions and measures can provide a basis and reference direction for the optimization and improvement of SMHR.

This research has several limitations that should be considered. Firstly, due to the subjective nature of HRM evaluation, subjective factors may inevitably influence the evaluation process of various factors. Moreover, the SMHR system evaluated in this study only focused on three aspects, namely, employee recruitment, employee welfare management, and employee training. The evaluation did not take into account the correlation effects of other departments related to HRM, which could limit the generalizability of the results. To address these limitations, future research could aim to gain a more comprehensive understanding of the process and principles of HRM. This would involve fully grasping relevant information and establishing a complete system that could make the entire evaluation process more fair and objective. Additionally, a more comprehensive analysis of the SMHR system of the enterprise should be conducted, taking into account the correlation effects of other departments. Such an approach could provide practical significance for the sustainable development of the enterprise.

## Supporting information

S1 Data (ZIP)
